# Integrating single-cell RNA-seq and spatial transcriptomics reveals MDK-NCL dependent immunosuppressive environment in endometrial carcinoma

**DOI:** 10.3389/fimmu.2023.1145300

**Published:** 2023-04-04

**Authors:** Xinnian Yu, Linjun Xie, Jianjuan Ge, Huixin Li, Shanliang Zhong, Xiaolin Liu

**Affiliations:** ^1^ Department of Internal Medicine, The Affiliated Cancer Hospital of Nanjing Medical University & Jiangsu Cancer Hospital & Jiangsu Institute of Cancer Research, Nanjing, China; ^2^ Department of Pharmacy, The Affiliated Cancer Hospital of Nanjing Medical University & Jiangsu Cancer Hospital & Jiangsu Institute of Cancer Research, Nanjing, China; ^3^ Department of Oncology, Affiliated Tumor Hospital of Nantong University, Nantong, China; ^4^ Department of Gynecology, The Affiliated Obstetrics and Gynecology Hospital of Nanjing Medical University & Nanjing Maternity and Child Health Care Hospital, Nanjing, China; ^5^ Center of Clinical Laboratory Science, The Affiliated Cancer Hospital of Nanjing Medical University & Jiangsu Cancer Hospital & Jiangsu Institute of Cancer Research, Nanjing, China; ^6^ Office of Ethics Committee, The Affiliated Cancer Hospital of Nanjing Medical University & Jiangsu Cancer Hospital & Jiangsu Institute of Cancer Research, Nanjing, China

**Keywords:** endometrial cancer, scRNA-seq, spatial transcriptome, tumor microenvironment, midkine

## Abstract

**Objectives:**

The tumor microenvironment (TME) play important roles in progression of endometrial carcinoma (EC). We aimed to assess the cell populations in TME of EC.

**Methods:**

We downloaded datasets of single-cell RNA-seq (scRNA-seq) and spatial transcriptome (ST) for EC from GEO, and downloaded RNA-Seq (FPKM) and clinical data of TCGA-UCEC project from TCGA. The datasets were analyzed using R software.

**Results:**

We obtained 5 datasets of scRNA-seq, 1 of ST and 569 samples of RNA-seq. Totally, 0.2 billion transcripts and 33,408 genes were detected in 33,162 cells from scRNA-seq. The cells were classified into 9 clusters, and EC cells were originated from epithelial cells and ciliated cells. Gene set variation analysis (GSVA) indicated that the pathways enriched in the subclusters of epithelial cells and endothelial cells were significantly different, indicating great heterogeneity in EC. Cell-cell communication analyses showed that EC cells emitted the strongest signals, and endothelial cells received more signals than other cells. Further analysis found that subclusters of 1 and 2 of epithelial cells were showed a more malignant phenotype, which may confer malignant phenotype to subcluster of 0 of endothelial cells through MK pathway by MDL-NCL signal. We also analyzed communications between spatial neighbors with ST data and confirmed the findings on MDL-NCL in cell-cell communication. TCGA and GEO analyses indicated that the expression levels of NCL was inversely correlated with ImmuneScore.

**Conclusion:**

Our study revealed EC cells can confer malignant phenotype to endothelial cells by MDK-NCL signal, and NCL is associated with suppressed immune activity. EC cells may shape TME by inhibiting immune cells and “educating” stromal cells *via* MDK-NCL signal.

## Introduction

Tumors are characterized by extensive heterogeneity which plays a critical role in tumor progression and treatment response (1). The tumor microenvironment (TME) consists of both malignant cells and stromal cells with different functional phenotypes and spatial distribution patterns (2, 3). Cancer cells are heterogeneous due to genetic diversification and clonal selection (1, 4). Stromal cells are the cells surrounding the tumor, such as immune cells, inflammatory cells, fibroblasts and endothelial cells (5). Even for one type of stromal cells, heterogeneity is still exist and they may be composed of several subpopulations exerting different biological roles (4). TME diversity is a challenge for the treatment of tumors, which will influence response to anti-cancer therapy (6). However, the extent of this heterogeneity as well as how the cells are shaped by other cells in the TME remains poorly known.

Endometrial carcinoma (EC) is the most common malignancy of the female reproductive system in the developed countries, where has the highest incidence (7, 8). The two most frequent types are endometrioid adenocarcinoma (EAC) and serous cystadenocarcinoma (SCC), which have different risk factors, prognosis, patterns of metastasis, and microscopic appearance (9, 10). Although surgery alone can cure most EC patients, the prognosis of patients with more aggressive variants of EC remains poor (11). EC has both inter- and intra-tumoral heterogeneity (12). A high proportion of ECs are composed of different tumor cell clones with different morphologic and molecular features. Tumor heterogeneity may have an important impact on diagnosis, prognosis, and therapeutic decisions. It is therefore important to identify minor cell subpopulations.

ScRNA-seq is a method to measure the expression levels of all genes over thousands to millions of individual cells, and reveals heterogeneity at cell level (13, 14). For example, Lambrechts et al. characterized the phenotype and co-optive behavior of stromal cells using scRNA-seq techniques (4). Therefore, with the aid of scRNA-seq, we can explore TME of EC to improve the understanding of the diagnosis, treatment and management of EC. In the present study, we investigated EC using the scRNA-seq data from GEO, and tried to uncover heterogeneity of EC. We also integrated scRNA-seq and spatial transcriptome (ST) to reveal tissue architecture of EC.

## Materials and methods

### Data collection

Single-cell RNA-seq (scRNA-seq) data for 5 patients with EAC (**Table S1**) and spatial transcriptomic data for one patient with EC (GSM6177623 in GSE203612) were downloaded from Gene Expression Omnibus (GEO).

We downloaded RNA-Seq (FPKM) and clinical data of TCGA-UCEC project from TCGA (https://portal.gdc.cancer.gov, accessed November 2022), and obtained 553 RNA-Seq files for EC tissues and 35 for normal endometrial tissues. We downloaded mRNA profiles of 145 EC patients from GEO (GSE120490) (15) and used as validation dataset.

### Single-cell transcriptome analysis

All analyses in the present study were performed using R software (version 4.1.1). The scRNA-seq data were analyzed using Seurat package (version 4.2) (16). Low-quality cells with less than 300 or over 7500 expressed genes, or over 25% unique molecular identifiers (UMIs) derived from the mitochondrial genome were removed. Then, mitocondrial, ribosomal and hemoglobin genes were removed from the data sets. Finally, 33,162 cells and 33,408 genes were retained for further analysis.

We used canonical correlation analysis (CCA) to correct for batch effects across datasets (17). Top 2000 highly variable genes were identified using FindVariableFeatures function in Seurat package with default parameters. Principal component analysis (PCA) were performed with the highly variable genes after Z-score normalization. Uniform manifold approximation and projection (UMAP) dimension reduction was performed with the top 30 significant principal components (PCs). Clusters were determined using the FindClusters function (resolution = 0.5). Marker genes for each of clusters were identified as those with a fold change larger than 2 using the Seurat FindMarkers function. The clusters were annotated to known cell types with the marker genes using CellMarker 2.0 (18) and PanglaoDB (19). MUC16/CA125 expression levels was used to identify malignant EC cells (20).

### Spatial transcriptome analysis

ST data were analyzed with Seurat package (version 4.2) (16) using similar method as scRNA-seq. The single cell data were predicted on the ST data with the FindTransferAnchors and TransferData functions from the Seurat package with default settings.

### Gene set variation analysis

Human gene sets from 50 hallmark pathways were retrieved using msigdbr package (version 7.5.1). Then we used method mentioned by Lambrechts et al. (4) to remove genes associated to two or more pathways, and applied GSVA using standard settings to assign pathway activity estimates to individual cells with GSVA package (version 1.40.0).

### Cell-cell communication analysis

We analyzed intercellular communications using CellChat package (version 1.5.0) (21) for scRNA-seq and NICHES package (version 1.0) (22) for ST.

### TCGA analysis

After log-normalizing the expression of each gene to an average expression of 1 in the samples of EM and removing outliers of the average expression of each marker gene, we used boxplots to evaluate per cell type the combined expression of marker genes for each subcluster. The survival analyses were conducted as previously described (23). Briefly, we separated the patients into two groups according to the expression levels of a gene. Then the overall survival (OS) was evaluated by the log-rank test with the survival package (R package version 3.4). We used cox proportional hazards model to calculate hazard ratios (HRs) and their 95% confidence intervals (CIs) and forestplot package (R package version 3.1) to draw forest plot. We also calculated ImmuneScore, StromalScore and ESTIMATEScore using estimate package (R package version 1.0.13). The relationships between the scores and the genes in MK pathway were evaluated using Spearman correlation test.

### Validation dataset analysis

To further validate the associations between ImmuneScore and the genes in MK pathway, we calculated the scores and their correlations with GSE120490 dataset by the method above mentioned.

### Tissue specimens

We collected 32 EC tissues and 19 normal endometrial tissues from Women’s Hospital of Nanjing Medical University and The Affiliated Cancer Hospital of Nanjing Medical University. The EC patients were not received any preoperative radiation, chemotherapy, or other anticancer therapies before surgery. This study was approved by the Ethics Committee of Women’s Hospital of Nanjing Medical University (No. 2021NFKSL-071). All subjects involved in this study signed informed consent documents prior to the operation.

### Real-time quantitative PCR

Total RNA was extracted and reversely transcribed as previously described (24). The expression levels of genes were detected with the primers in **Table S2**. β-action was used as a reference gene. All samples were analyzed in duplicate for each gene. The differences between groups were assessed using Mann-Whitney test.

## Results

### scRNA-seq and cell typing of EC

**Figure 1:**
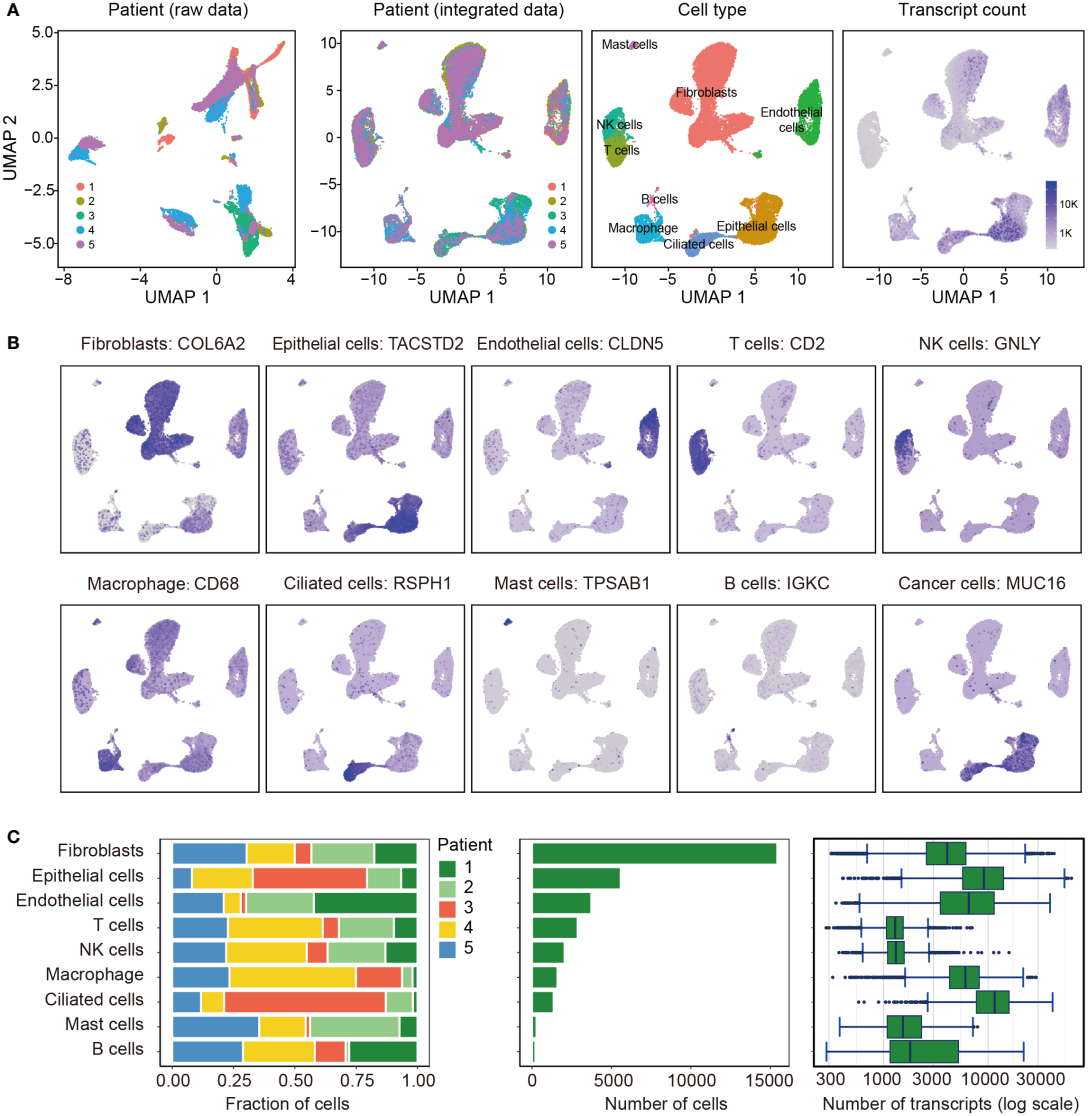
Overview of the 33,162 single cells from endometrioid carcinoma. **(A)** Uniform manifold approximation and projection (UMAP) of the 33,162 cells profiled here, with each cell color coded for (left to right): the corresponding patient in raw data and integrated data, the associated cell type and the number of transcripts (UMIs) detected in that cell (log scale as defined in the inset). K, thousand. **(B)** Expression of the representative marker genes for the cell types. **(C)** For each of the 9 cell clusters (left to right): the fraction of cells originating from each of the 5 patients, the number of cells and box plots of the number of transcripts.

All 5 tissues subjected to scRNA-seq were from patients with endometrioid carcinoma. Totally, 0.2 billion UMIs and 33,408 genes were detected in 33,162 cells after quality filtering. We found several clusters were composed one sample, suggesting there were potential batch effects (**Figure 1A**), thus we corrected the batch effects using CCA. These cells were classified into 9 clusters, and their marker genes are shown in **Table S3**. According to these marker genes, the 9 clusters were assigned to known cell lineages, including fibroblasts, epithelial cells, endothelial cells, T cells, NK cells, macrophage, ciliated cells, mast cells and B cells (**Figure 1A**). **Figure 1B** shows the expression levels of a representative marker gene for each cell type. We found that the EC cells were originated from epithelial cells and ciliated cells according to the expression of MUC16/CA125, and cancer cells were detected more transcripts than other cells (**Figures 1A, C**, **Table S4**).

### Subclusters of endothelial cells

**Figure 2:**
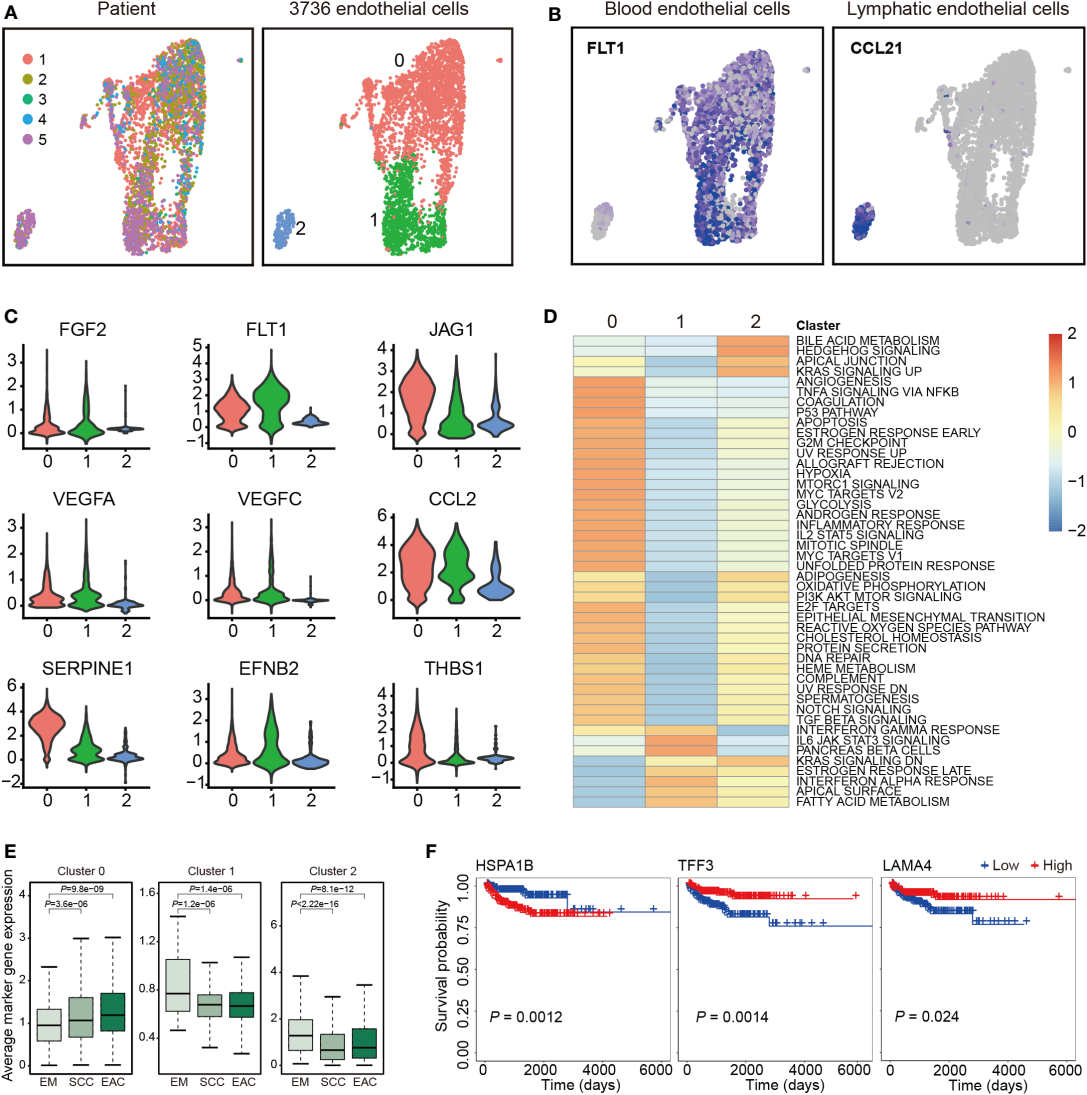
Endothelial cell clusters. **(A)** Uniform manifold approximation and projection (UMAP) plot of 3,736 endothelial cells, color-coded by their corresponding patient (left) or associated cluster (right). **(B)** UMAP plot color-coded for expression (blue to gray) of marker genes for blood and lymphatic endothelial cells. **(C)** Violin plots of selected genes involved in angiogenesis. **(D)** Differences in pathway activities scored per cell by GSVA between the different clusters. Shown are *t* values from a linear model. **(E)** Average expression of the marker genes for endothelial cells from each cluster in TCGA samples from endometrium (EM, n = 35), serous cystadenocarcinoma (SCC, n = 133) or endometrioid adenocarcinoma (EAC, n = 401). **(F)** The three marker genes associated with overall survival of endometrial carcinoma patients in TCGA.

Totally, 3,736 endothelial cells were detected and re-clustered into 3 clusters (**Figure 2A**). Based their marker genes (**Table S5**), clusters 0 and 1 were assigned as blood endothelial cells and cluster 2 were assigned as lymphatic endothelial cells (**Figure 2B**). Further analysis showed that selected genes associated with angiogenesis were highly expressed in clusters 0 and 1 (**Figure 2C**). Pathway analysis showed a significant phenotypic diversity among the three clusters, and cluster 0 was involved in more pathways than the other two pathways (**Figure 2D**). We analyzed expression of the marker genes in TCGA and found the marker genes from cluster 0 had a higher expression levels in SCC and EAC than those in EM, however, the marker genes from clusters 1 and 2 showed an opposite tendency (**Figure 2E**). Survival analysis with the marker genes showed that HSPA1B, TFF3 and LAMA4 were associated with EC survival (**Figure 2F**).

### Subclusters of epithelial cells

**Figure 3:**
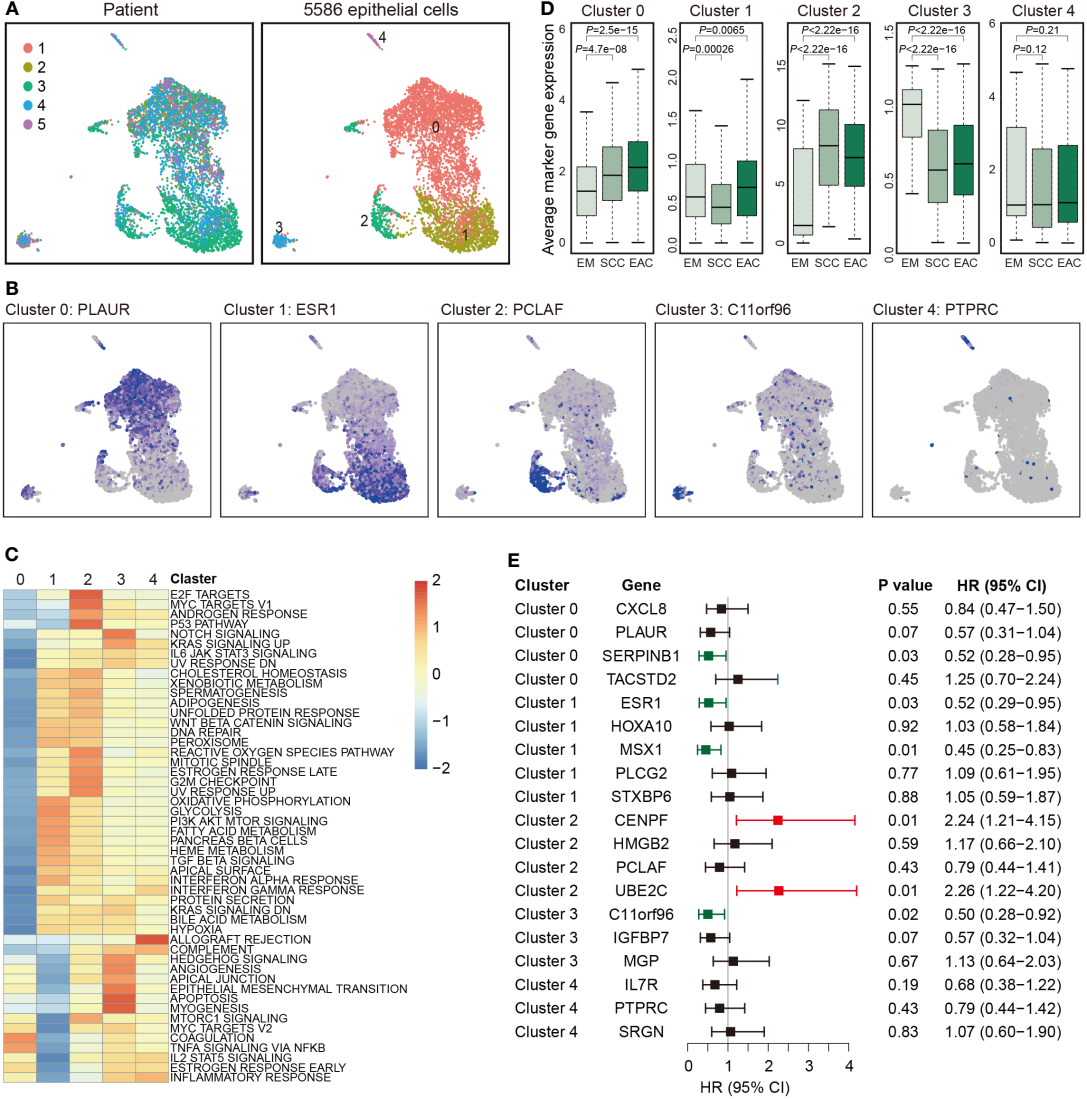
Epithelial cell clusters. **(A)** Uniform manifold approximation and projection (UMAP) plot of 5,586 epithelial cells, color-coded by their corresponding patient (left) or associated cluster (right). **(B)** Expression of represent marker genes for each cluster. **(C)** Differences in pathway activities scored per cell by GSVA between the different clusters. Shown are *t* values from a linear model. **(D)** Average expression of the marker genes for endothelial cells from each cluster in TCGA samples from endometrium (EM, n = 35), serous cystadenocarcinoma (SCC, n = 133) or endometrioid adenocarcinoma (EAC, n = 401). **(E)** Forest plot showing HR (95% CI) of the marker genes in TCGA.

Totally, 5,586 epithelial cells were detected and re-clustered into 5 clusters (**Figure 3A**). The marker genes for the clusters are listed in **Table S6** and representative marker genes were showed in **Figure 3B**. To characterize functions of these clusters, we compared pathway activities. Cluster 0 was much different from the other four clusters. Clusters 1 and 2, as well as clusters 3 and 4 were similar (**Figure 3C**). The average expression of the marker genes in TCGA are presented in **Figure 3D**. The marker genes of all clusters but not cluster 4 showed a significant association between EAC and EM or between SCC and EM. Survival analysis with the marker genes are showed in **Figure 3E**, and six genes were associated with EC survival (**Figure 3E**).

### Cell-cell communication between cells in scRNA-seq data

**Figure 4:**
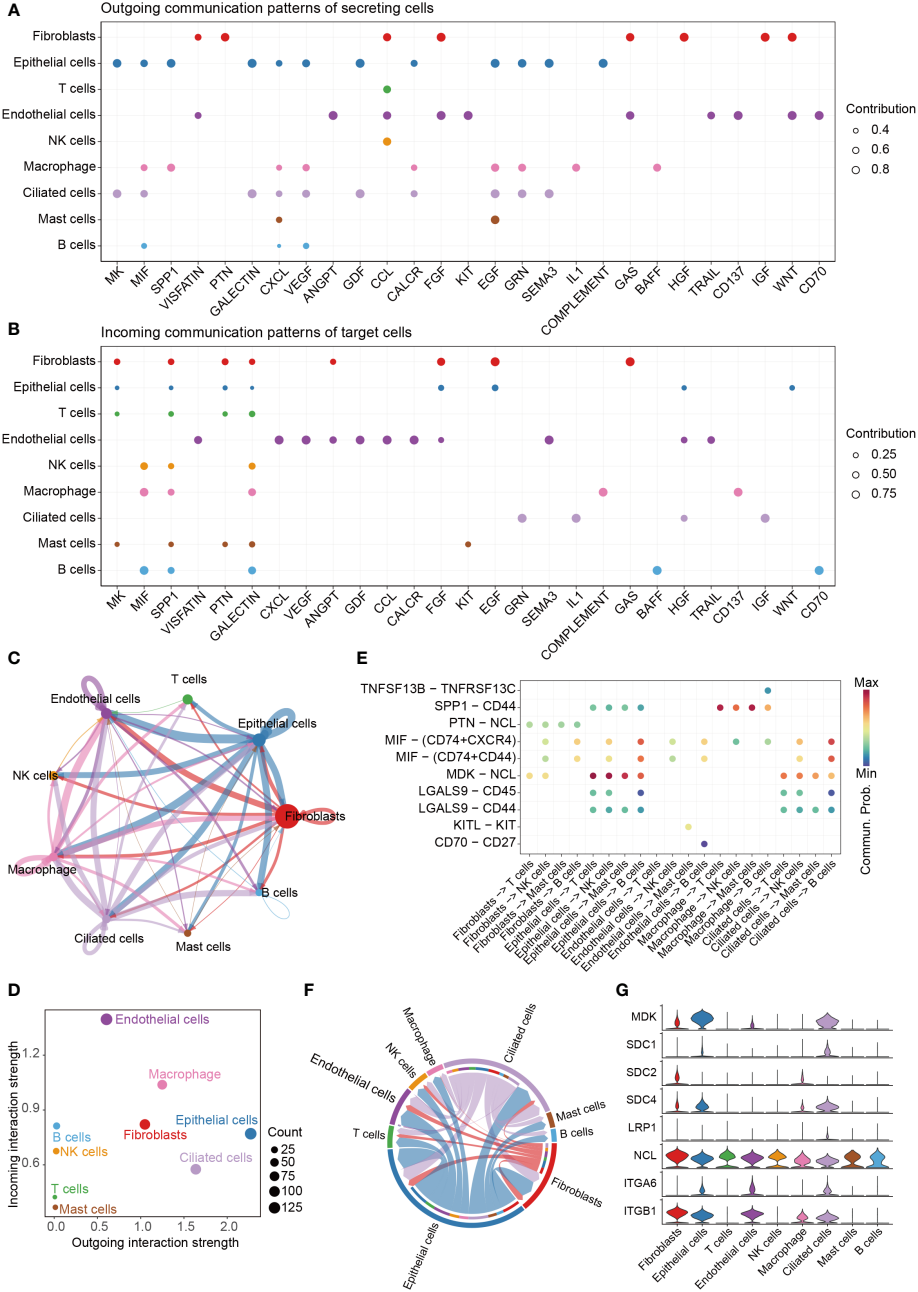
Cell-cell communication. **(A)** The dot plot showing the comparison of outgoing signaling patterns of secreting cells. **(B)** The dot plot showing the comparison of incoming signaling patterns. **(C)** Circle plot showing the communication strength between interacting cells. **(D)** Outgoing and incoming interaction strength of the cells. **(E)** Comparison of the significant ligand-receptor pairs between cells, which contribute to the signaling from fibroblasts, epithelial cells, endothelial cells, macrophage and ciliated cells to T cells, NK cells, Mast cells and B cells. **(F)** Chord plot showing inferred intercellular communication network of MK signaling. **(G)** Violin plots showing the expression of the 8 genes involved in MK signaling network.

We analyzed the communications between the 9 cell clusters using CellChat with scRNA-seq data. The cells were interacted with each other through 27 pathways (**Figures 4A, B**). The pathways in **Figures 4A, B** were ordered by their strength (Left to right), and the top one pathway was MK pathway. Epithelial cells emitted the strongest signals (**Figures 4A, C, D**, **S1**); and endothelial cells received more signals than other cells (**Figures 4B, D**). We found the cells were mainly contacted through Ligand-Receptor (L-R) pairs of MDK – NCL in MK signal pathway (**Figures 4E**, **S2**). The signals in MK signal pathway are showed in **Figure 4F**, and the expression levels of the ligands and receptors in MK pathway are showed in **Figure 4G**. Midkine (MK, MDK) was mainly expressed in epithelial cells and ciliated cells, and nucleolin (NCL) was expressed in all 9 type of cells.

### Interaction between epithelial cells and endothelial cells

We further analyzed the communications between subclusters of epithelial cells and endothelial cells. We found cluster 1 of epithelial cells (Ep. 1) emitted the strongest signals, and cluster 0 of endothelial cells (En. 0) received more signals than other clusters (**Figures S3A–C**). MK was also the top one pathway involved in the communication between epithelial cells and endothelial cells. MDK – NCL was still the top one L-R pairs (**Figures S3D**).

### Niche interactions

**Figure 5:**
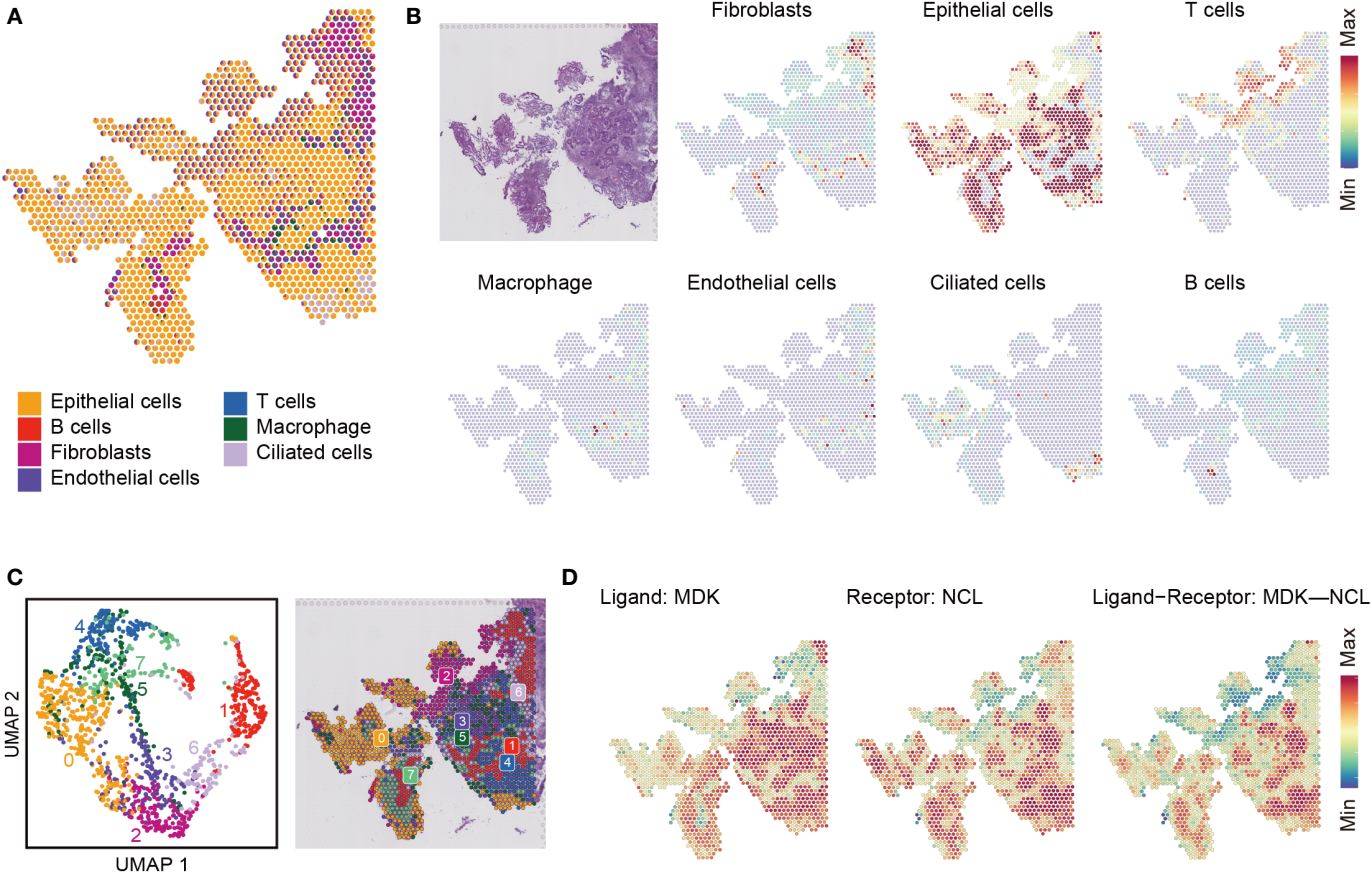
Cell type mapping on endometrial carcinoma tissue slice. **(A)** Spatial scatter pie plot representing the proportions of the cells from the reference atlas within capture locations in the endometrial carcinoma tissue. **(B)** Predicted proportion within each capture location for the cell types. **(C)** Unbiased clustering of spatial transcriptomic (ST) spots (left) and local microenvironment estimated from ST dataset by limiting cell-cell interactions to those within local neighborhoods, yielding a ‘niche’ atlas for each transcriptomic spot (right). **(D)** MDK-NCL signaling atlas.

We also analyzed the communications between spatial neighbors using NICHES with ST data. Totally, 6.6 million UMIs and 33,538 genes were detected in 1,351 spots. In one spot, about 4,911 UMIs and 2,403 unique genes were detected. We analyzed and integrated ST data with the scRNA-seq datasets, and 7 type of cells detected in scRNA-seq were mapped to EC tissue slice (**Figures 5A, B**). We used spatial scatter pie plot to show the distributions and proportions of the 7 type of cells (**Figures 5A**), and epithelial cells were the predominant cells. Then we investigated cellular niche using NICHES, which estimates local microenvironment in ST data. We could see, from UMAP plot in **Figure 5C**, some notable overlaps between the microenvironments of the 8 clusters, and the overlaps implied existence of interactions between them. We calculated row sums for all L-R pairs in “NeighborhoodToCell” assay, and then order the sums decreasingly. The top 20 L-R pairs are listed in **Figure S4**. We found MDK was presented in 5 of the top 20 L-R pairs, including MDK-NCL. We further plotted MDK, NCL as well as niche interactions of MDK-NCL to the tissue regions (**Figure 5D**).

### Immunosuppressive environment induced by MDK – NCL pathway

**Figure 6:**
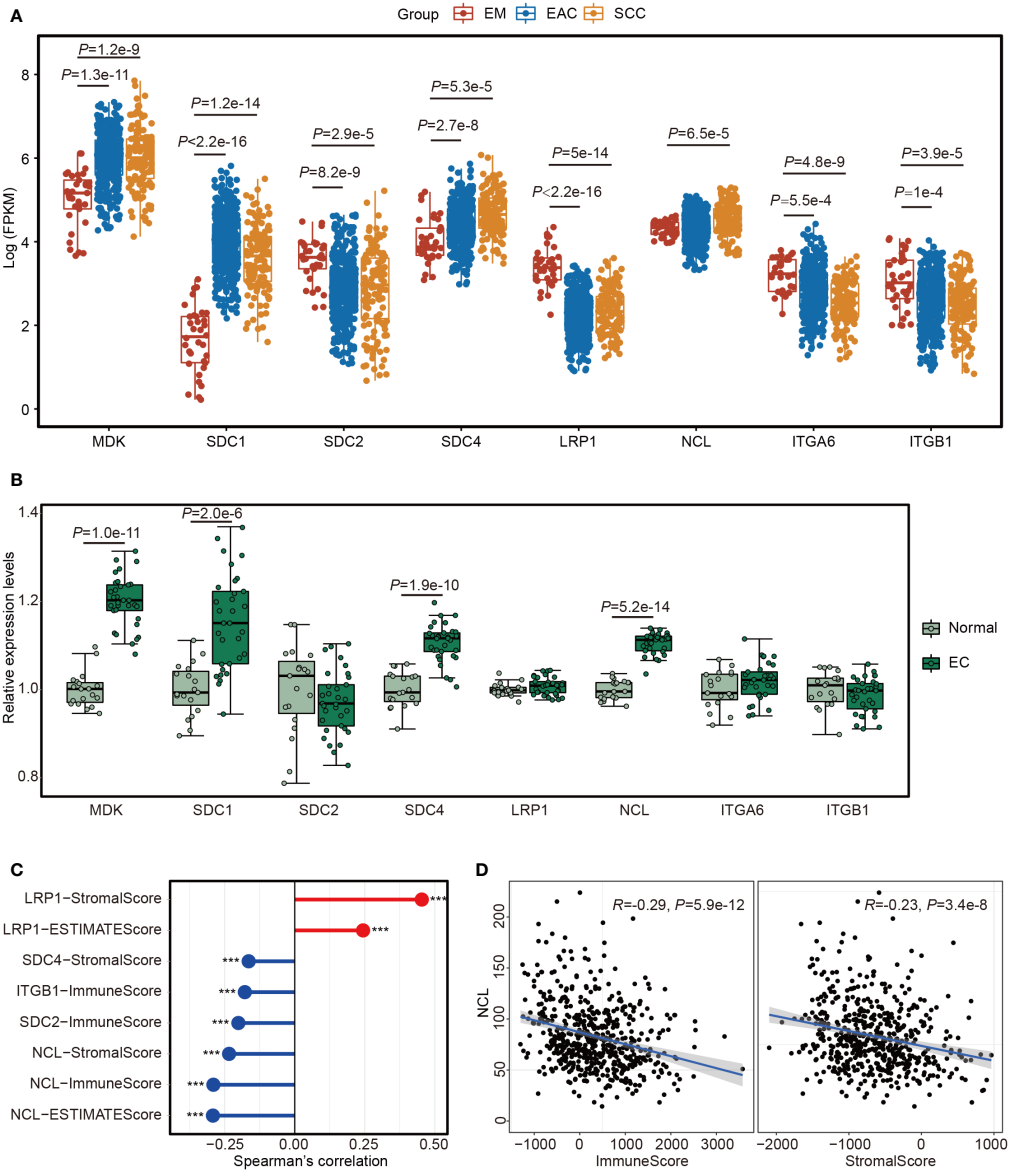
The expression of the 8 genes involved in MK signaling network. **(A)** Boxplot showing the expression levels of the 8 genes in TCGA samples from endometrium (EM, n = 35), serous cystadenocarcinoma (SCC, n = 133) or endometrioid adenocarcinoma (EAC, n = 401). **(B)** Boxplot showing the expression levels of the 8 genes in 32 endometrial carcinoma (EC) tissues and 19 normal endometrial tissues. **(C)** Lollipop chart showing the significant associations between the 8 genes and the scores estimated by “estimate” package with the TCGA data. ***P<0.001. **(D)** Scatter plots showing the Spearman’s correction between NCL and ImmuneScore or StromalScore.

To explore the roles of MK pathway in EC, we analyzed the expression levels of the ligands and receptors in this pathway with the data from TCGA. All ligands and receptors were differentially expressed between SCC and EM or between EAC and EM (**Figure 6A**). MDK had a higher expression level in SCC and EAC than EM. NCL only had a slight difference between SCC and EM. In our cohort, both MDK and NCL had higher expression levels in EC tissues than in normal tissues (**Figure 6B**).

We calculated ImmuneScore, StromalScore and ESTIMATEScore using estimate package with TCGA cohort and assessed their relationships with the genes in MK pathway. The significant associations are showed in **Figure 6C**. We noted that most genes showed a reverse correlation with the scores (**Figures 6C**) and NCL was inversely correlated with all three scores (**Figures 6D**). After analyzing GSE120490, we further confirmed the findings in TCGA dataset (**Figures S5**), thus EC cells may suppress immune cell responses in TME by MK pathway through MDK-NCL signaling.

## Discussion

In the present study, we analyzed scRNA-seq data for EC and presented the transcriptional and regulatory landscape of EC at single-cell resolution, revealing great heterogeneity in both cancer cells and stromal cells.

In our study, the cells were classified into 9 clusters with a slightly different from the original study by Regner et al. (20). We found the five datasets had potential batch effects, because several clusters were from one patients. However, Regner et al. did not correct the batch effects, which may contribute to the difference. We found the cancer cells from the five EC patients were derived originally from epithelial cells and ciliated cells. More transcripts were detected in cancer cells than other cells, suggesting high activity of cancer cells. In the original study by Regner et al. (20), all five EC patients were diagnosed as EAC, which accounts for approximately 80% of endometrial epithelial malignancies (25). The patients 3 had the highest proportion of cancer cells, and the patients 1 had the lowest proportion, showing an inter-tumoral heterogeneity in EC. Besides malignant ciliated cells, the malignant epithelial cells were further classified into 5 clusters, showing an intra-tumoral heterogeneity in EC. With regard to stromal cells, we re-clustered endothelial cells into 3 clusters, two clusters of blood endothelial cells and one cluster of lymphatic endothelial cells, indicating a heterogeneity in stromal cells of EC.

Cancer cells communicate with surrounding cells mainly by MK signal pathway in the TME of EC. MDK, a heparin-binding growth factor, promotes cell growth, survival, migration, angiogenesis, cytokine expression, differentiation and other activities of target cells (26, 27). In the present study, scRNA-seq showed that MDK was highly expressed in epithelial cells and ciliated cells, and TCGA indicated that MDK had a higher expression level in SCC and EAC than EM, thus MDK may be served as a biomarker for EC. Further analyses showed that MDK-NCL was the top one L-R pairs involved in the communication in TME of EC. NCL is one the most abundant proteins of the nucleolus and plays a central role in polymerase I transcription (28, 29). NCL is also found in the nucleoplasm, cytoplasm and on the cell membrane (30, 31). At the cell membrane, NCL was found to interact with several ligands involved in cell proliferation, apoptosis and angiogenesis (29). We found the expression levels of NCL was inversely correlated with both ImmuneScore and StromalScore, suggesting EC cells may inhibit immune cells in the TME by MDK-NCL signal. We also found Ep. 1 and Ep. 2 emitted the strongest signals, and En. 0 received more signals than other clusters. The pathway analyses showed that En. 0 as well as Ep. 1 and Ep. 2 were involved in more pathways than other clusters. Ep. 1 and Ep. 2 may more malignant than other subclusters of epithelial cells. En. 0 may be educated by Ep. 1 and Ep. 2 and acquired malignant phenotype. ST data further confirmed the signal of MDK-NCL in EC. It has been shown that MDK-NCL was associated with immune environment and progression of tumors. For example, MDK was reported to reconstruct immunosuppressive environment in melanoma and gallbladder cancer (32, 33). MDK was found to support progression of gastric cancer (34), and increased EGFR signaling under hypoxia through interaction with NCL (35). Taken together, EC cancer cells may shape TME by inhibiting immune cells and “educating” stromal cells *via* MDK-NCL signal.

There are several limitations of the present study. First, because of lacking enough reference data for EC, the subclusters of epithelial cells and endothelial cells were unable to annotate to known cell types. Second, we did not validate the roles of MDK-NCL signal in EC with *in vitro* or *in vivo* experiments. Third, we only included 32 EC tissues and 19 normal endometrial tissues, and the sample size was relative small. Further studies should validate our results in larger sample of patients, and confirm the roles of MDK-NCL signal with both *in vitro* and *in vivo* experiments.

In conclusion, our study revealed tumoral heterogeneity in the cellular composition and molecular phenotype of the TME in EC. EC cells can confer malignant phenotype to endothelial cells by MDK-NCL signal, and NCL is associated with suppressed immune activity. EC cells may shape TME by inhibiting immune cells and “educating” stromal cells *via* MDK-NCL signal. Blocking MDK-NCL signal might help to inhibit the progression of EC. Our results provided new potential targets for EC therapy.

## Data availability statement

The datasets presented in this study can be found in online repositories. The names of the repository/repositories and accession number(s) can be found within the article/**Supplementary Materials**.

## Ethics statement

The studies involving human participants were reviewed and approved by Ethics Committee of Women’s Hospital of Nanjing Medical University. The patients/participants provided their written informed consent to participate in this study.

## Author contributions

SZ and XL designed the study. SZ, XY, JG, XL and LX analyzed the data. SZ and HL wrote the manuscript. All authors contributed to the article and approved the submitted version.

## Supplementary material

Supplementary Figure 1 The outgoing signals from each cell type. The line width repents the strength of the signal.

Supplementary Figure 2 MDK-NCL transfers the strongest signal. **(A)** Comparison of the significant ligand-receptor pairs between cells, which contribute to the signaling from fibroblasts, epithelial cells, macrophage and ciliated cells to T cells, endothelial cells, NK cells, Mast cells and B cells. **(B)** Comparison of the significant ligand-receptor pairs between cells, which contribute to the signaling from epithelial cells and ciliated cells to fibroblasts, T cells, endothelial cells, NK cells, macrophage, Mast cells and B cells. **(C)** Contribution of each Ligand-Receptor (L-R) pairs in the communication between the cells.

Supplementary Figure 3 Interaction between epithelial cells and endothelial cells. **(A)** Outgoing and incoming interaction strength of the subclusters of epithelial cells and endothelial cells. En. 0–En. 2, subclusters 0–2 of endothelial cells; and Ep. 0–Ep. 4, subclusters 0–4 of epithelial cells; **(B)** Heatmaps showing outgoing and incoming interaction strength as well as involved pathways. **(C)** Contribution of each Ligand-Receptor (L-R) pairs in the communication between the subclusters of epithelial cells and endothelial cells.

Supplementary Figure 4 Signaling atlas of the top 20 Ligand-Receptor pairs.

Supplementary Figure 5 Validation of MK signaling in GSE120490. **(A)** Lollipop chart showing the significant associations between the 8 genes and the scores estimated by “estimate” package. *, *P*<0.05; **, *P*<0.01; ***, *P*<0.001. **(C)** Scatter plots showing the Spearman’s corrections between NCL/MDK and ImmuneScore.
