# Supplementary material for: Integrating single-cell RNA-seq and spatial transcriptomics reveals MDK-NCL dependent immunosuppressive environment in endometrial carcinoma
Source: Front Immunol. 2023 Apr 4;14:1145300. doi: 10.3389/fimmu.2023.1145300 (PMC10110842; doi:10.3389/fimmu.2023.1145300)
Supplement: Supplementary Figure 1 — The outgoing signals from each cell type. The line width repents the strength of the signal. [file DataSheet_1.pdf]

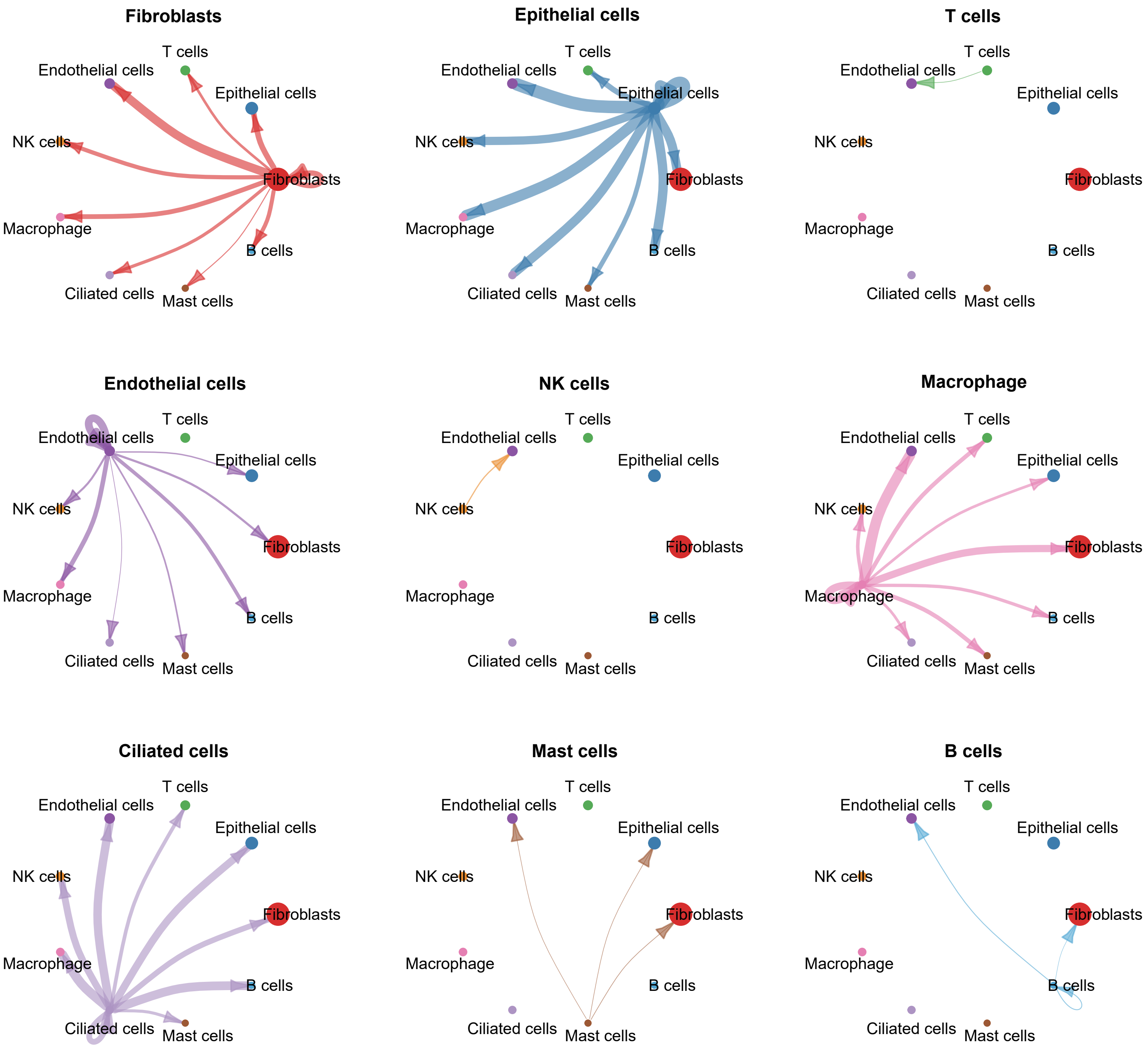
Fibroblasts
T cells
Endothelial cells
Epithelial cells
NK cells
Fibroblasts
Macrophage
B cells
Ciliated cells
Mast cells
Epithelial cells
T cells
Endothelial cells
Epithelial cells
NK cells
Fibroblasts
Macrophage
B cells
Ciliated cells
Mast cells
T cells
T cells
Endothelial cells
Epithelial cells
NK cells
Fibroblasts
Macrophage
B cells
Ciliated cells
Mast cells
Endothelial cells
T cells
Endothelial cells
Epithelial cells
NK cells
Fibroblasts
Macrophage
B cells
Ciliated cells
Mast cells
NK cells
T cells
Endothelial cells
Epithelial cells
NK cells
Fibroblasts
Macrophage
B cells
Ciliated cells
Mast cells
Macrophage
T cells
Endothelial cells
Epithelial cells
NK cells
Fibroblasts
Macrophage
B cells
Ciliated cells
Mast cells
Ciliated cells
T cells
Endothelial cells
Epithelial cells
NK cells
Fibroblasts
Macrophage
B cells
Ciliated cells
Mast cells
Mast cells
T cells
Endothelial cells
Epithelial cells
NK cells
Fibroblasts
Macrophage
B cells
Ciliated cells
Mast cells
B cells
T cells
Endothelial cells
Epithelial cells
NK cells
Fibroblasts
Macrophage
B cells
Ciliated cells
Mast cells
