# Supplementary figures and images for: Integrating single-cell RNA-seq and spatial transcriptomics reveals MDK-NCL dependent immunosuppressive environment in endometrial carcinoma
Source: Front Immunol. 2023 Apr 4;14:1145300. doi: 10.3389/fimmu.2023.1145300 (PMC10110842; doi:10.3389/fimmu.2023.1145300)

a

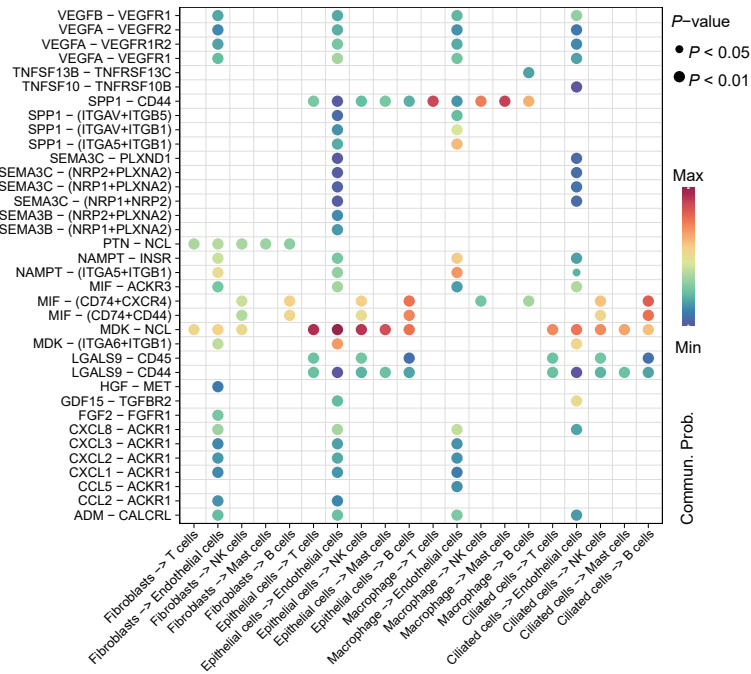

b

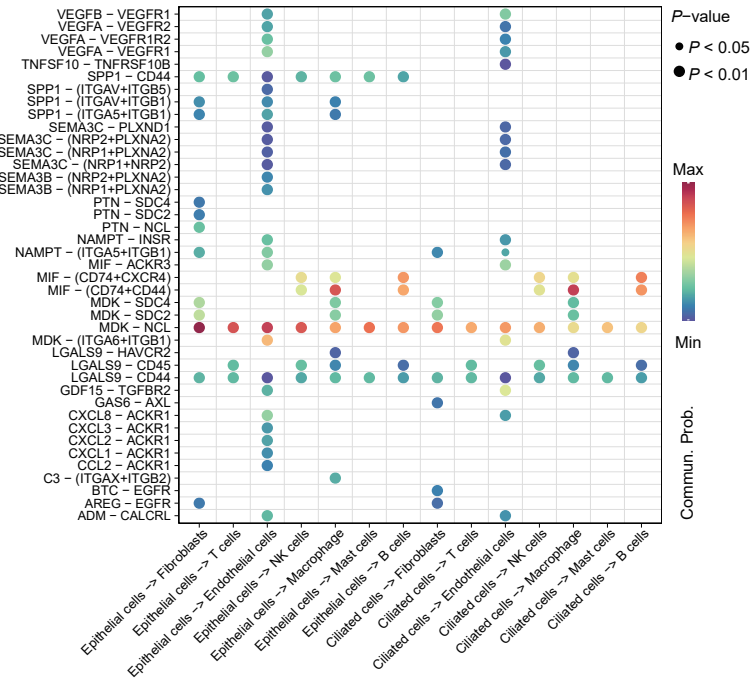

c

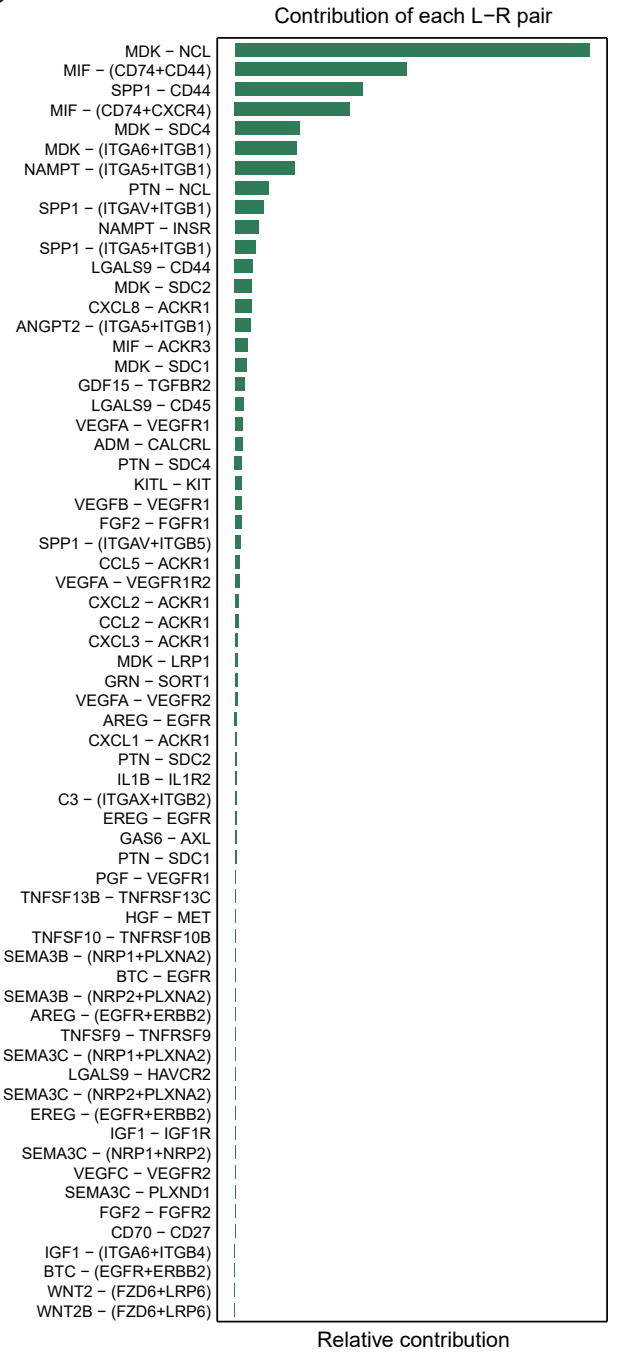

Supplement: Supplementary Figure 2 — MDK-NCL transfers the strongest signal. (A) Comparison of the significant ligand-receptor pairs between cells, which contribute to the signaling from fibroblasts, epithelial cells, macrophage and ciliated cells to T cells, endothelial cells, NK cells, Mast cells and B cells. (B) Comparison of the significant ligand-receptor pairs between cells, which contribute to the signaling from epithelial cells and ciliated cells to fibroblasts, T cells, endothelial cells, NK cells, macrophage, Mast cells and B cells. (C) Contribution of each Ligand-Receptor (L-R) pairs in the communication between the cells. [file DataSheet_2.pdf]

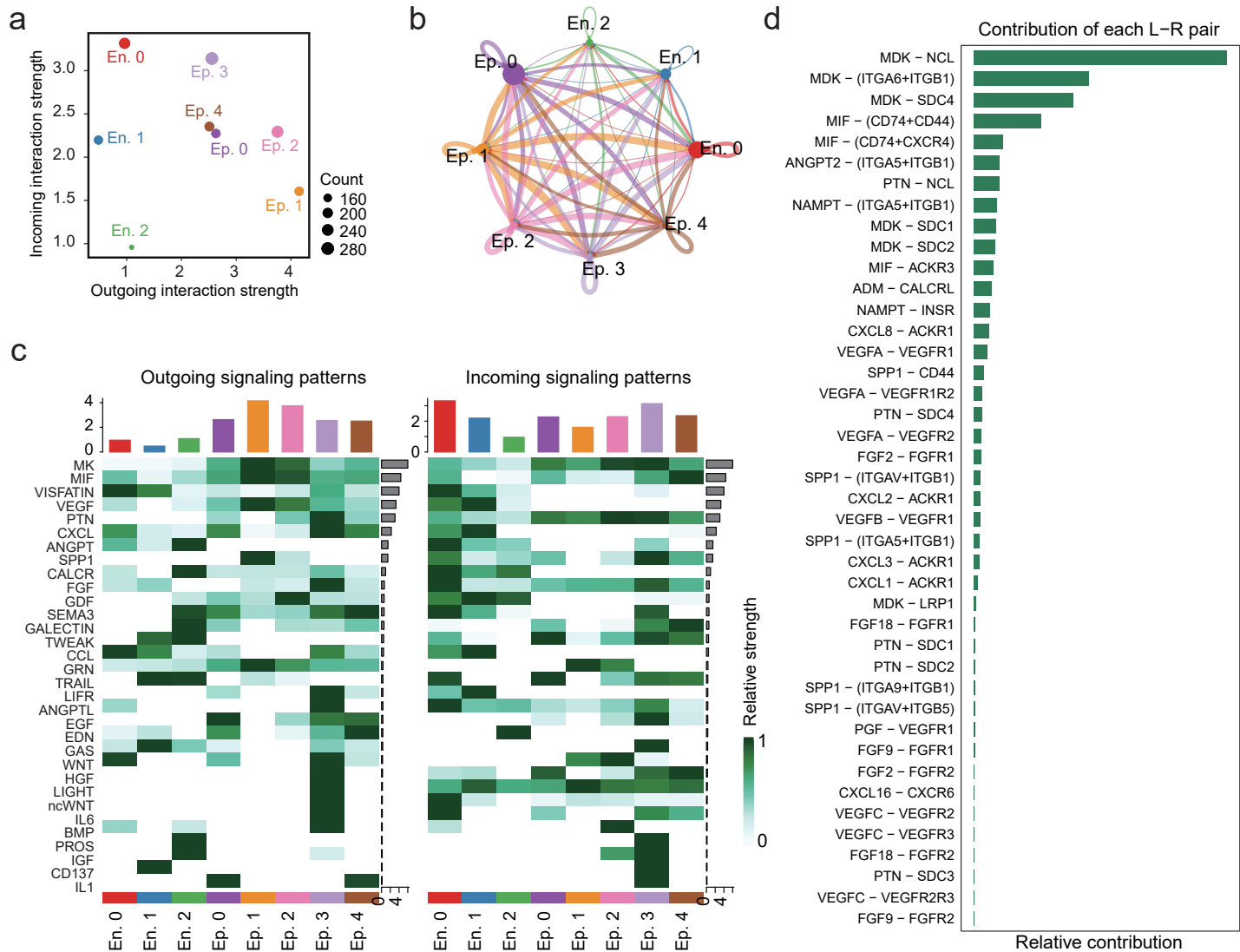

Supplement: Supplementary Figure 3 — Interaction between epithelial cells and endothelial cells. (A) Outgoing and incoming interaction strength of the subclusters of epithelial cells and endothelial cells. En. 0–En. 2, subclusters 0–2 of endothelial cells; and Ep. 0–Ep. 4, subclusters 0–4 of epithelial cells; (B) Heatmaps showing outgoing and incoming interaction strength as well as involved pathways. (C) Contribution of each Ligand-Receptor (L-R) pairs in the communication between the subclusters of epithelial cells and endothelial cells. [file DataSheet_3.pdf]

a

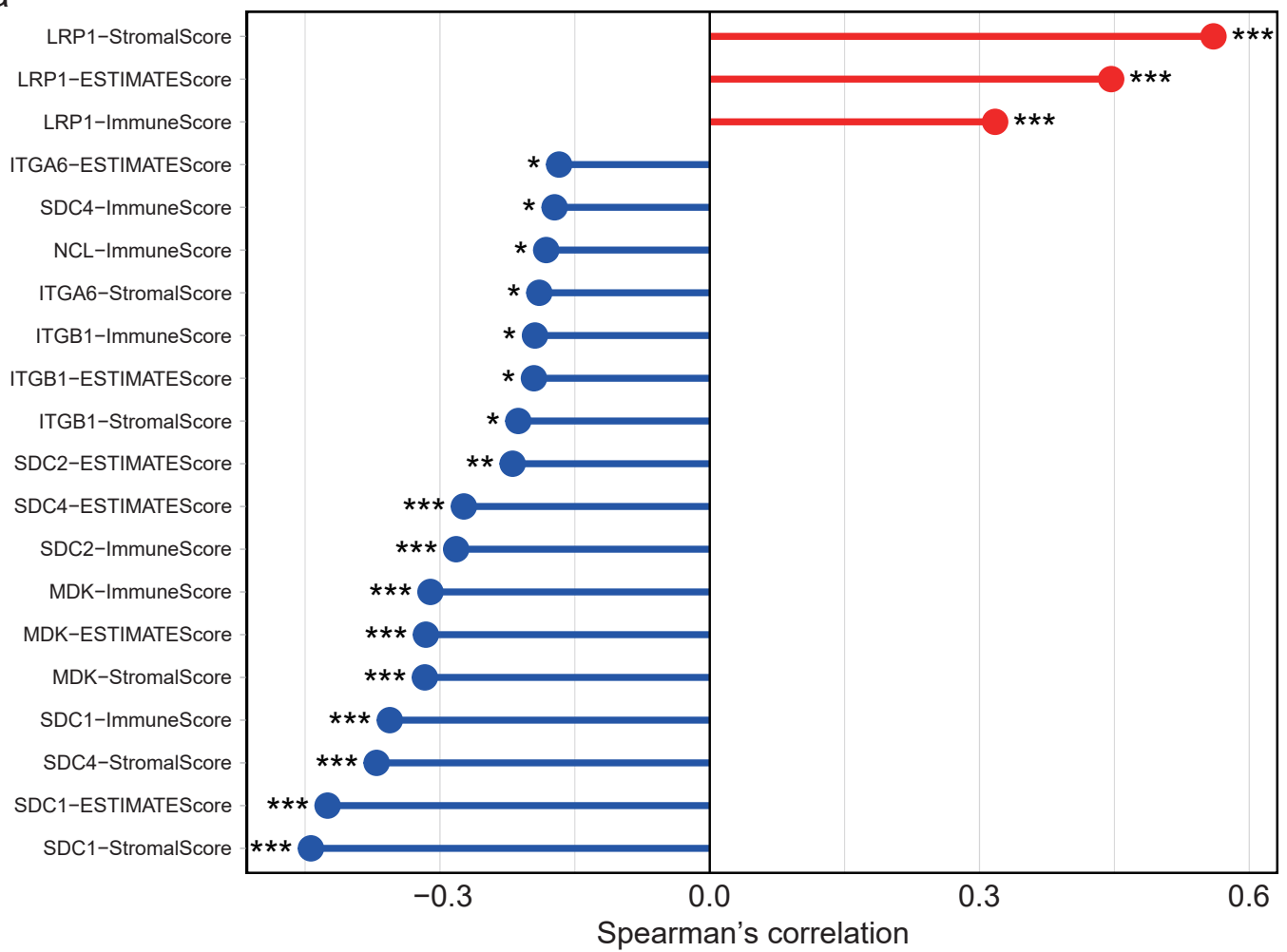

b

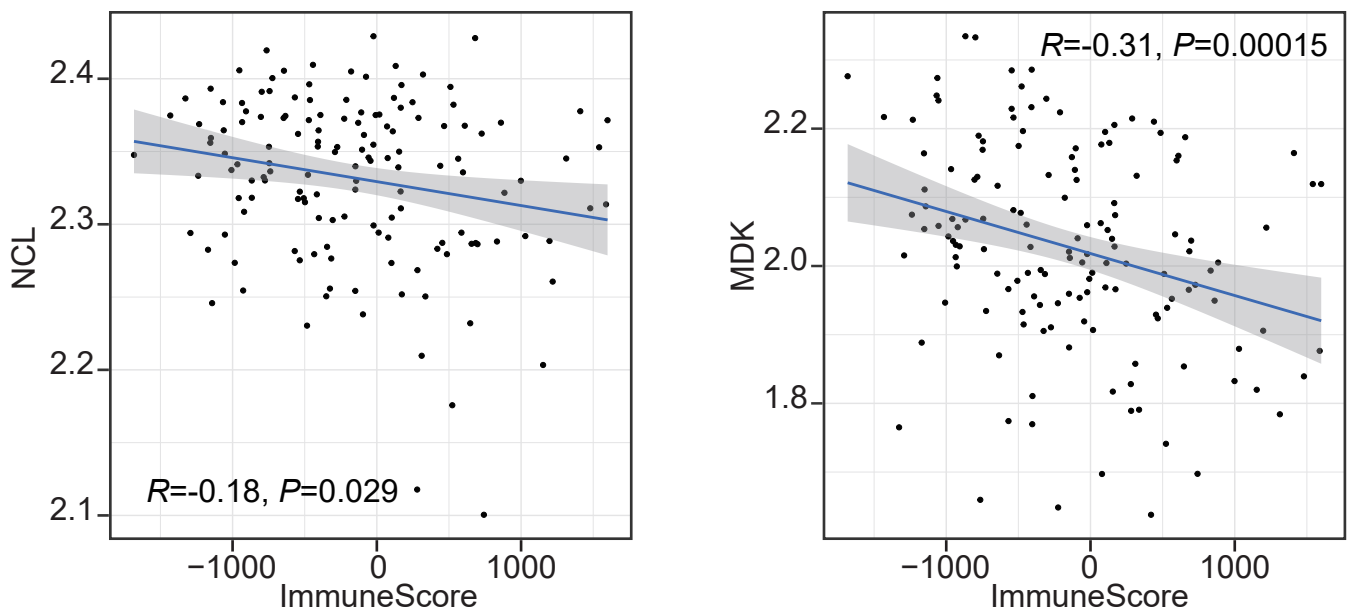

Supplement: Supplementary Figure 5 — Validation of MK signaling in GSE120490. (A) Lollipop chart showing the significant associations between the 8 genes and the scores estimated by “estimate” package. *, P<0.05; **, P<0.01; ***, P<0.001. (C) Scatter plots showing the Spearman’s corrections between NCL/MDK and ImmuneScore. [file DataSheet_5.pdf]
