# Supplementary material for: Integrating single-cell RNA-seq and spatial transcriptomics reveals MDK-NCL dependent immunosuppressive environment in endometrial carcinoma
Source: Front Immunol. 2023 Apr 4;14:1145300. doi: 10.3389/fimmu.2023.1145300 (PMC10110842; doi:10.3389/fimmu.2023.1145300)
Supplement: Supplementary Figure 4 — Signaling atlas of the top 20 Ligand-Receptor pairs. [file DataSheet_4.pdf]

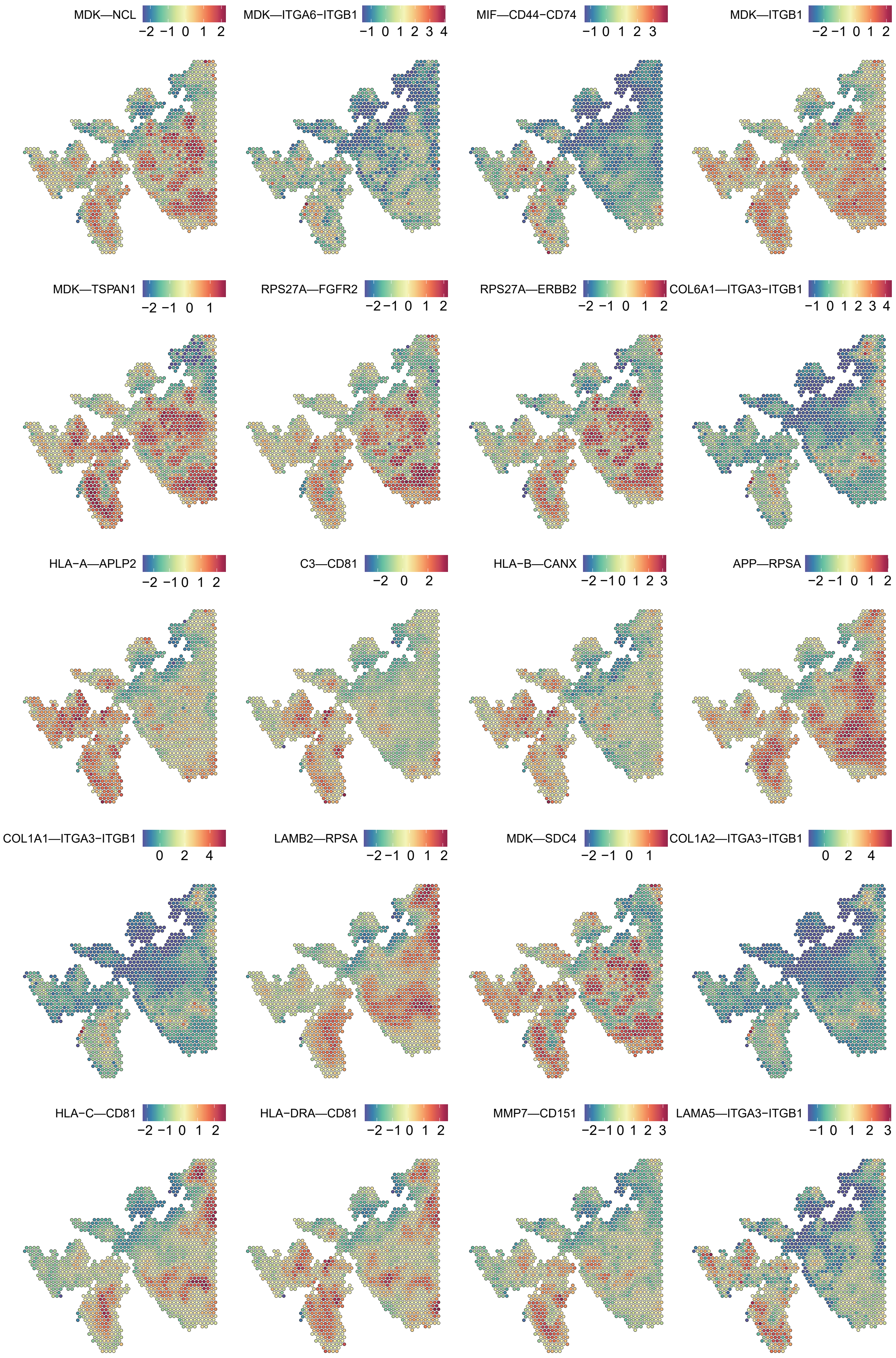

MDK—NCL
−2 −1 0 1 2
MDK—ITGA6−ITGB1
−1 0 1 2 3 4
MIF—CD44−CD74
−1 0 1 2 3
MDK—ITGB1
−2 −1 0 1 2
MDK—TSPAN1
−2 −1 0 1
RPS27A—FGFR2
−2 −1 0 1 2
RPS27A—ERBB2
−2 −1 0 1 2
COL6A1—ITGA3−ITGB1
−1 0 1 2 3 4
HLA−A—APLP2
−2 −1 0 1 2
C3—CD81
−2 0 2
HLA−B—CANX
−2 −1 0 1 2 3
APP—RPSA
−2 −1 0 1 2
COL1A1—ITGA3−ITGB1
0 2 4
LAMB2—RPSA
−2 −1 0 1 2
MDK—SDC4
−2 −1 0 1
COL1A2—ITGA3−ITGB1
0 2 4
HLA−C—CD81
−2 −1 0 1 2
HLA−DRA—CD81
−2 −1 0 1 2
MMP7—CD151
−2 −1 0 1 2 3
LAMA5—ITGA3−ITGB1
−1 0 1 2 3
